# Polymorphism of tumor necrosis factor alpha (TNF-alpha) gene promoter, circulating TNF-alpha level, and cardiovascular risk factor for ischemic stroke

**DOI:** 10.1186/1742-2094-9-235

**Published:** 2012-10-10

**Authors:** Guanglin Cui, Haoran Wang, Rui Li, Lina Zhang, Zongzhe Li, Yan Wang, Rutai Hui, Hu Ding, Dao Wen Wang

**Affiliations:** 1 Departments of Internal Medicine and Institute of Hypertension, Tongji Hospital, Tongji Medical College, Huazhong University of Science & Technology, 1095# Jiefang Ave, Wuhan, 430030, China; 2 Fuwai Hospital, Peking Union Medical College and Chinese Academy of Medical Sciences, Beijing, China

**Keywords:** Tumor necrosis factor alpha (TNF-α), Genetics, Cardiovascular risk factors, Function study

## Abstract

**Background:**

Tumor necrosis factor-α (TNF-α) is one of the most typical pro-inflammatory cytokines with both beneficial and destructive properties for the central nervous system. Increasing evidences have demonstrated the important role of TNF-α in the development of ischemic stroke, but studies examining the possible association with stroke or direct functional effects of polymorphisms in TNF-α have been contradictory.

**Findings:**

In this study, a 2-kb length of the proximal promoter of the TNF-α was screened and four polymorphisms were investigated in the case–control study. Our data confirmed the association between -308G/A variant with stroke in 1,388 stroke patients and 1,027 controls and replicated in an independent population of 961 stroke patients and 821 controls (odds ratio (OR) = 1.34, 95% confidence interval (CI) =1.02 to 1.77 and OR = 1.56, 95% CI = 1.09 to 2.23, respectively). To reconcile the association between polymorphisms and stroke and to give a comprehensive picture of the genetic architecture of this important gene, we performed a meta-analysis of 15 published studies in an Asian population. Our results demonstrated an association between rs1800629 and ischemic stroke (OR = 1.43, 95% CI = 1.21 to 1.69). Another meta-analysis results of 14 studies demonstrated that ischemic stroke patients have higher serum TNF-α level than the control subjects (standardized mean difference (SMD) = 2.33, 95% CI = 1.85 to 2.81). *In vitro* evaluation of potential interaction between variants of the TNF-α gene (−308G/A, -857C/T, and -1031T/C) demonstrated that these three polymorphisms could interact together to determine the overall activity of the TNF-α gene.

**Conclusions:**

These findings strongly implicate the involvement of TNF-α in the pathogenesis of stroke.

## Introduction

Inflammation has important roles in the development and rupture of atherosclerotic lesions leading to cardiovascular disease events [1]. The studies have shown that cerebral ischemia and inflammation are closely inter-related: ischemia is a robust stimulus for the potential damaging inflammation, while infection as well as its associated inflammation is an important risk factor for ischemic stroke [2,3]. Furthermore, recent studies have shown that the genetic variation in tumor necrosis factor-α (TNF-α), IL-6, and other pro-inflammatory cytokines might increase the risk for development of vascular dementia and lacunar infarction [4,5].

TNF-α is one of the most typical pro-inflammatory cytokines with both beneficial and destructive properties for the central nervous system [6,7]. TNF-α effects on lipid metabolism, coagulation, and endothelial function, and the increasing release of TNF-α may contribute to the odds of ischemic stroke patients [8]. Several functional polymorphisms in the promoter region of the gene coding for TNF-α have been studied in different groups respectively, but these experimental results are inconsistent [9-11]. Recently a meta-analysis showed the absence of any significant functional association between TNF-α production and SNP genotypes or heliotype combinations of the TNF-α gene promoter in healthy subjects in combined analysis of Caucasians and non-Caucasians [12]. However, these results may have different effects in the white subjects and the Asian population [13], and these differences may be representative of gender, social background, and geographical variation [14].

There is increasing evidence that inflammatory variables are associated with atherogenesis and predict risk of cardiovascular disease [15]. Several studies have reported elevations of pro-inflammatory cytokines in peripheral blood [16-18] as well as in cerebrospinal fluid in patients with ischemic stroke [19,20]. However, higher circulating levels of TNF-α might be expected to be associated with increased stroke risks based on experimental evidence [21,22], but some epidemiologic evidence is of no significance [14,23]. Thus, it remains uncertain whether the polymorphisms of TNF-α promoter play a direct physiological role in regulating serum TNF-α concentration and if the TNF-α gene promoter functional polymorphisms can interact together to determine the overall activity of the TNF-α gene promoter is still not completely understood. Besides, if these TNF-α promoter functional polymorphisms could potentially contribute to the etiology of stroke in the Asian population is still needed to be illuminated.

In this study, we investigated the relationship between common variants in TNF-α gene and TNF-α level, cardiovascular risk factors, and stroke in a large collaborative analysis of Chinese Han populations, to explore the randomized allocation of alleles to better understand the nature of the association between TNF-α and stroke.

## Methods

### Study populations

In the initial study, we investigated the association between common variants in TNF-α gene and stroke using a case–control cohort that has been described previously [24]. In brief, stroke patients (*n* = 1,388) were recruited between November 2004 and January 2009 from five hospitals in Wuhan, China, according to the *International Classification of Diseases (9th Revision, codes 430–438*). Ethnically and geographically matched controls (*n* = 1,027) were randomly recruited from the population by a house-to-house recruitment protocol. All control subjects were free of neurological conditions and followed the same exclusion criteria as cases. For the purpose of replication, we introduced the second independent case–control cohort that has been fully described elsewhere [24] and comprised 961 stroke patients and 821 controls. The diagnostic criteria for stroke and the recruited criteria for controls were identical to those used in the first study. In addition, there were no overlapping participants between these two studies. All patients and controls were carefully matched by geographic region of recruitment, were of Han Chinese ancestry.

A total of 376 healthy controls (mean age 56 ±12 years; 145 men) were selected for serum TNF-α concentration determination. They were recruited randomly from the general population in Dongxi Lake community and all the subjects were free of cardiovascular disease. Additional exclusion criteria were referred to those reported previously [10]. All the study protocols were approved by the Review Board of the Ministry of Public Health, Ministry of Science and Technology of China, and the ethics committees at all participating hospitals and informed written consents were obtained from all participants.

### Resequencing and genotyping analysis

A 2-kb length of the human TNF-α gene promoter region between −2,000 and +30 base pairs (GenBank Accession: NG_007462) were identified by direct sequencing of genomic DNA derived from 96 randomly selected individuals from the control samples. Amplification and primers used for genetic variation screening are available in Additional file 1: Table S1. Fluorescent dye-terminator cycle sequencing was performed and products were analyzed with an Applied Biosystems 3130xl capillary sequencer (Applied Biosystems Inc., Foster City, CA, USA). The Chromas program (Technelysium Pty. Ltd., Helensvale, Queensland, Australia) was used to identify putative polymorphisms that were then confirmed by two independent observers. We further confirmed polymorphism positions and individual genotypes by reamplifying and resequencing these loci from the opposite strand.

An ABI PRISM 7900HT Sequence Detection System (Applied Biosystems Inc., Foster City, CA, USA) was used for genotyping by TaqMan-based assays. TaqMan probes and PCR primers are available from the Additional file 1: Table S2. Genotypes were verified by sequencing 50 of each genotype for common variants, and all heterozygotes and homozygotes for less common variants. All the DNA samples for cases and control subjects were run in the same batch.

### Serum TNF-α level measurement

Serum samples were collected after overnight fasting and stored at −80°C. TNF-α levels in serum were determined in 376 unrelated healthy individuals by OPTEIA ELISA kit (BD Biosciences, San Jose, CA), according to the manufacturer’s instructions. All samples were analyzed in duplicate and recombinant standards were included on every plate. The arithmetic mean of the duplicate samples was considered for analysis. The intra-assay coefficient of variation was 1.9 ± 0.8% and inter-assay variation was 2.7 ± 1.2%.

### DNA constructs

To study the functionality of the polymorphisms in the promoter region of TNF-α, 10 different constructs were made. A 1,607 base pairs fragment was amplified by PCR from DNA from one individual with no variant in this promoter region using Oligonucleotides was as follows: forward primer, 5’ GGCCAGATCTCGACCAGAGCCCCACACG 3’; reverse primer, 5’ GGGGAAGCTTGGCTGAGGAACAAGCACCG 3’. Oligonucleotides were designed with BglII and GGCC tail at the 5’ end of the forward oligonucleotide and HindIII and GGGG tail at the 5’ end of the reverse oligonucleotide. PCR products were TA-cloned into pMD® 18-T vector system following the manufacturer’s conditions (Takara Biotechnology, Dalian, China). This construct was then served as the template to generate other constructs that contains different mutations in the promoter region. Mutations were generated through overlap PCR with specially designed oligonucleotides. All the promoter fragments were subsequently cloned into pGL3 basic vector (Promega, Madison, USA) through double digested ligation reaction. These constructs were finally confirmed by sequencing with Applied Biosystems 3130xl capillary sequencer (Applied Biosystems Inc., Foster City, CA, USA). All the oligonucleotides which were used to create the site mutation were listed in the Additional file 1: Table S3.

### Transient transfection and luciferase assay in cultured HepG2 cells

The human hepatoblastoma cells (HepG2) were cultured in 100 mm dishes in Dulbecco’s modified Eagle’s medium supplemented with 10% fetal calf serum (FCS).

HepG2 cells in 96-well format (4 × 10^4^ cells/well) were transiently transfected with 20 ng of the appropriate plasmids or control vector, 1 ng of pRL-TK co-transfector, and 0.5 μL of Lipofectamine 2000 (Invitrogen, Carlsbad, CA, USA), and the cells were lysed 48 h after transfection. Luciferase activity was assayed using the Dual-Luciferase® Reporter (DLR^TM^) Assay System (TM040, Promega, Madison, WI, USA). Firefly luciferase expression levels were adjusted with reference to Renilla luciferase activity. Three independent experiments were performed for each reporter.

### Statistical analyses

The distributions of quantitative variables were tested for normality by use of a 1-sample Kolmogorov-Smirnov test. A χ^2^ test was used to test for qualitative variables, genotype/allele frequencies, and for the Hardy-Weinberg equilibrium (HWE) of the variant. Differences of quantitative variables between groups were analyzed using the Student’s t-test. Logistic regression models were performed to estimate ORs and 95% CI for the association between genotypes and stroke. The Bonferroni correction method was applied for correction for multiple testing.

Because observed TNF-α level was not normally distributed, to test for association between the polymorphisms within TNF-α gene and TNF-α level, statistical significance was calculated using the Mann–Whitney U test for non-parametric variables. Due to the small number of individuals homozygous for the less frequent allele, homozygotes have been pooled with the corresponding heterozygotes for the statistical analysis.

We divided the distributions of the TNF-α level among control subjects into three quartiles: Q1 (*n* = 125) represents the lower quartile, Q2 (*n* = 125) represents the moderate quartile, and Q3 (*n* = 126) represents the higher quartile. For the TNF-α promoter variant, serum TNF-α level, and traditional cardiovascular risk factor including age, sex, BMI, blood pressure, total cholesterol, and HDL cholesterol, unadjusted mean values of traditional cardiovascular risk factor with their respective standard deviations were obtained for each genotype. Mean differences in the traditional cardiovascular risk factor for each genotype and quartile were calculated.

One-way ANOVA was used to compare the relative luciferase activities of the different polymorphisms of TNF-α promoter. All statistics was performed with the SPSS 13.0 package (SPSS Inc., Chicago, IL, USA). A value of *P* <0.05 was taken as significant (two-tailed).

### Meta-analysis

The publications included in the analysis were selected from PubMed, Google Scholar, and from http://www.cnki.net/index.htm with the keywords ‘tumor necrosis factor alpha or TNF-α’, ‘stroke’, ‘TNF-α level and stroke’, ‘TNF-α polymorphism and stroke’, ‘cytokine polymorphism and stroke’, ‘inflammatory factor’, and the specific names and abbreviations of each gene. The analyzed data cover all English and Chinese publications from April 2000 to March 2012.

Meta-analysis was carried out using the Stata software 10.0 (STATA Corp, College Station, TX, USA) and the Q statistic was calculated to test for heterogeneity followed by calculation of I^2^ (percentage of effect size attributable to heterogeneity) [25]. Results from allele-based dominant model logistic regression analyses in individual studies of rs1800629 were meta-analyzed using a conservative random-effects pooling method (DerSimonian-Laird). A pooled SMD, together with 95% CI, was used for this meta-analysis. The SMD was chosen because the blood lipids were measured using different scanners [26]. The random-effects model was also used for this SMD meta-analysis. Begg’s funnel plots and Egger’s linear regression [27] were used to assess evidence for publication bias. Considering a wide variety of study designs among selected studies, sensitivity analyses were conducted as well.

## Results

### Localization and frequencies

In this study, the region from +30 to −2000 base pairs of the human TNF-α gene promoter has been analyzed by direct DNA sequencing. A total of eight polymorphisms located at positions −308, -238, -857, -863, -986, -1031, -1376, and −1671 were identified in the Chinese Han population. All polymorphisms were in Hardy-Weinberg equilibrium in our sample. The distribution of the genotypes and the relative allele frequencies is shown in Additional file 1: Table S4. The TNF-α promoter sequenced variants spanned the range of allele frequencies from rare (minor allele frequency [MAF] <0.01) to common (MAF >0.02) variants. Of the eight variants, we found one novel and rare variant -1376T/C. Moreover, rs1800630 polymorphism was in strong linkage disequilibrium (LD) with rs1799964. However, all other promoter polymorphisms analyzed in this study were not in significant LD with one another (Additional file 1: Figure S1).

### Association between variants at the promoter of TNF-α locus, cardiovascular risk factors, and stroke

**Figure 1:**
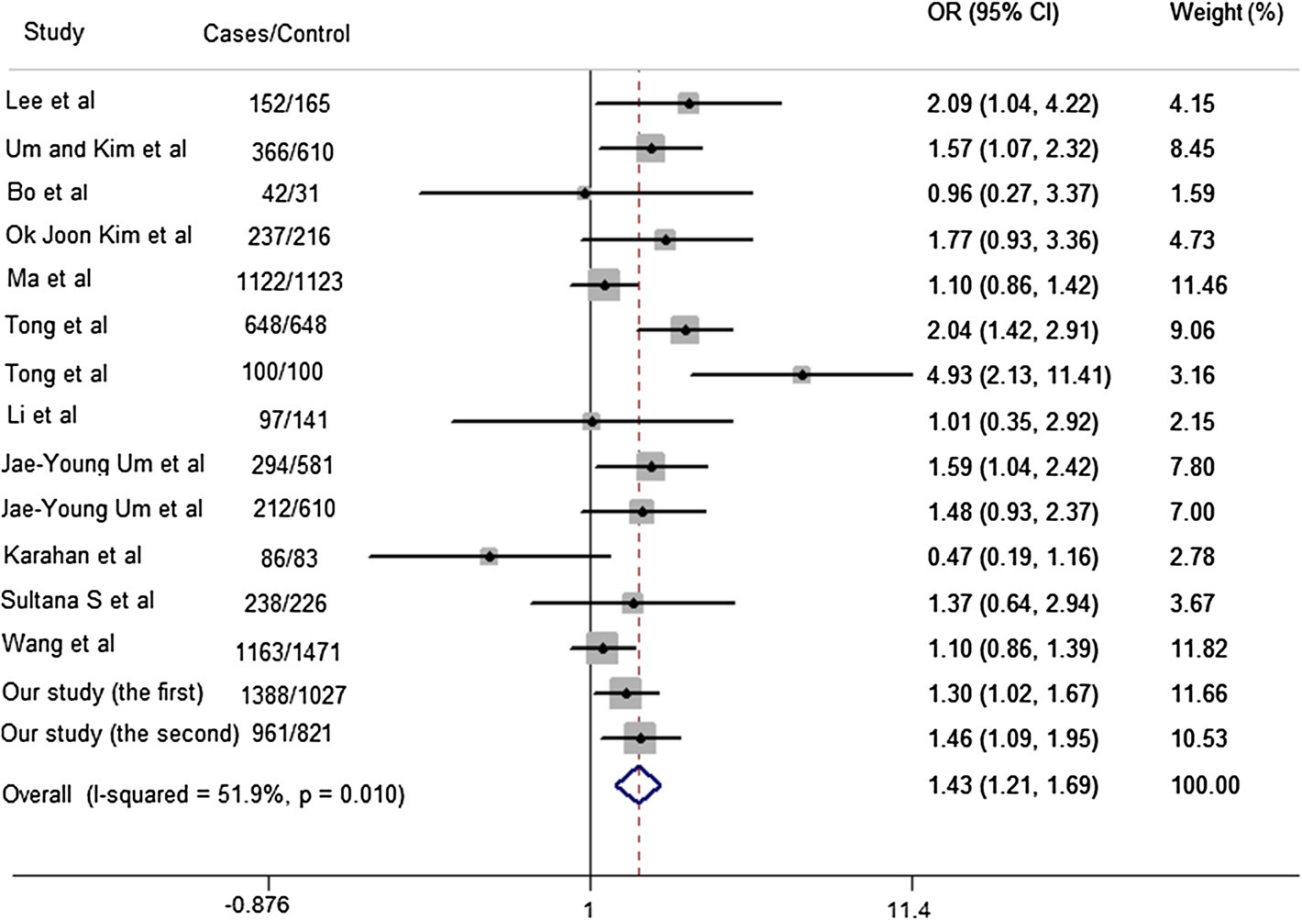
**Meta-analysis of rs1800629 and ischemic stroke association in Asian populations.** Gray squares indicate the OR, with the size of the square inversely proportional to its variance, and horizontal lines represent 95%CIs. The pooled results are indicated by the unshaded black diamond. All studies were conducted under a random-effects model.

Of the eight variants, four polymorphisms which minor allele frequencies (MAF >0.02) were selected for genotyping in the case–control study. The frequencies of these four tested variants did not deviate significantly from the HWE in cases and control subjects (all *P* >0.05). Two single nucleotide polymorphisms (SNPs) (rs1800629 and rs1799964) were significantly associated with increased risk of stroke in the first study independent of traditional cardiovascular risk factors in dominant models (OR = 1.34, 95% CI = 1.02 to 1.77, and OR = 1.25, 95% CI = 1.04 to 1.49, respectively). The significant genetic associations were observed under dominant genetic model in the second study for the SNP rs1800629 (adjusted OR = 1.56, 95% CI = 1.09 to 2.23), but this was not replicated for rs1799964 (adjusted OR = 1.02, 95% CI = 0.79 to 1.33) (Table 1). Because of the controversy results between rs1800629 and ischemic stroke in different ethnic communities [13], a meta-analysis was conducted in the Asian population. The characteristics of all participants from 13 studies are listed in Additional file 1: Table S5. A total of 15 studies including our two studies involving 14,959 individuals (7,106 cases and 7,853 control subjects) were analyzed in the next meta-analysis and our results demonstrated an association between rs1800629 and ischemic stroke (OR = 1.43, 95% CI = 1.21 to 1.69) under random effects model (Figure 1). The test of heterogeneity was significant (I^2^ = 51.9%, *P* = 0.01). No publication bias was found by using asymmetry analysis in the funnel plot (Additional file 1: Figure S2A), by Egger’s test (*P* = 0.698). Sensitivity analyses showed that the pooled effect estimates were positive in spite of omitting any one of selected studies, which indicated that there was no significant trend that the overall result was influenced by any individual study (Additional file 1: Figure S3A).

**Table 1:** Association between promoter variants with TNF-α promoter and ischemic stroke

**ID**	**SNP rs ID**	**Function**	**Population**	**MAF**	***P***_***allele***_	**MM**	**Mm**	**mm**	**Crude**	**Crude ORs (95%CI)**	**Adjusted**	**Adjusted ORs (95%CI)**
	**(M > m)**					***n*, (%)**	***n*, (%)**	***n*, (%)**	***P***_***dominant***_	**MM vs.Mm + mm(ref)**	***P***_***dominant***_	**MM vs.Mm + mm(ref)**
First study												
1	rs1799964	Promoter	Control (*n* = 1,027)	0.231	0.01	599 (58.3)	381 (37.1)	47 (4.6)	0.025	1.21 (1.02-1.42)	0.015	1.25 (1.04-1.49)
	(T > C)			IS (*n* = 1,388)	0.201		872 (62.8)	475 (34.2)	41 (3.0)				
2	rs1800629	Promoter	Control (*n* = 1,027)	0.071	0.026	886 (86.3)	136 (13.2)	5 (0.5)	0.034	1.30 (1.02-1.67)	0.038	1.34 (1.02-1.77)	
	(G > A)		IS (*n* = 1,388)	0.055		1,237 (89.1)	148 (10.7)	3 (0.2)					
3	rs1799724	Promoter	Control (*n* = 1,027)	0.173	0.45	700 (68.2)	299 (29.1)	28 (2.7)	0.15	0.88 (0.74-1.05)	0.81	0.98 (0.81-1.18)	
	(C > T)		IS (*n* = 1,388)	0.181		907 (65.3)	459 (33.1)	22 (1.6)					
4	rs361525	Promoter	Control (*n* = 1,027)	0.03		968 (94.3)	56 (5.5)	3 (0.2)	0.96	1.01 (0.71-1.43)	0.103	1.41 (0.93-2.13)	
	(A > G)		IS (*n* = 1,388)	0.029	0.781	1,309(94.3)	78 (5.6)	1 (0.1)					
Second study													
1	rs1799964	Promoter	Control (*n* = 821)	0.176	0.912	557 (67.8)	239 (29.1)	25 (3.1)	0.96	0.99 (0.81-1.21)	0.87	1.02 (0.79-1.33)	
	(T > C)		IS (*n* = 961)	0.177		650 (67.6)	281 (29.2)	30 (3.2)					
2	rs1800629	Promoter	Control (*n* = 821)	0.07	0.011	710 (86.5)	107 (13)	4 (0.5)	0.011	1.46 (1.09-1.96)	0.016	1.56 (1.09-2.23)	
	(G > A)		IS (*n* = 961)	0.05		868 (90.3)	90 (9.4)	3 (0.3)					
3	rs1799724	Promoter	Control (*n* = 821)	0.196	0.506	529 (64.4)	262 (31.9)	30 (3.7)	0.29	1.11 (0.91-1.35)	0.052	0.79 (0.62-1.00)	
	(C > T)		IS (*n* = 961)	0.187		642 (66.8)	278 (28.9)	41 (4.3)					
4	rs361525	Promoter	Control (*n* = 821)	0.037	0.974	761 (92.7)	59 (7.2)	1 (0.1)	0.95	0.99 (0.69-1.41)	0.22	0.74 (0.46-1.19)	
	(A > G)		IS (*n* = 961)	0.037		890 (92.6)	71 (7.4)	0 (0)					

IS, ischemic stroke; M, major allele; m, minor allele; MAF, minor allele frequency.

*P*_*allele*_ value of allele and Crude odds ratio (95% confidence interval) were determined by a 95% two-sided χ^2^ test, IS *versus* controls.

*P*_*dominant*_ value and adjusted odds ratio (95% confidence interval) were computed with multivariate unconditional logistic regression analysis by adjusting for gender, age, body mass index, hypertension, diabetes, hyperlipidemia, and smoking status.

**Figure 2:**
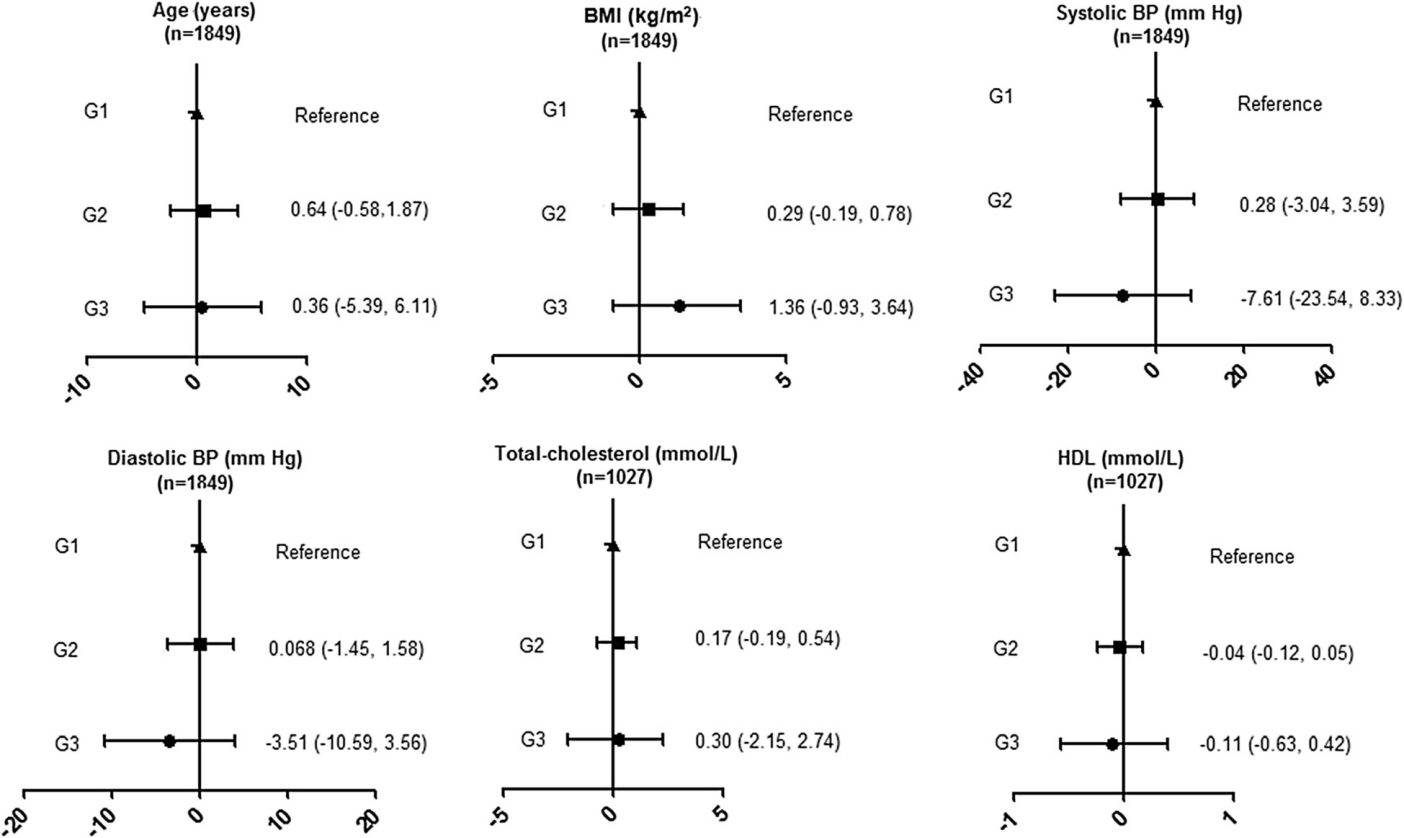
**Mean difference (95%CI) in cardiovascular traits by rs1800629 genotype.** BMI, body mass index; BP, blood pressure; G1, Homozygous common-allele; G2, Heterozygous; G3, Homozygous rare-allele.

We also investigated the association between SNP rs1800629 which with the largest effect on stroke in the meta-analysis with traditional cardiovascular risk factors. We observed no effect of the rs1800629 variant on any of the traditional cardiovascular risk factors (Figure 2).

### TNF-α variants and TNF-α levels

**Figure 3:**
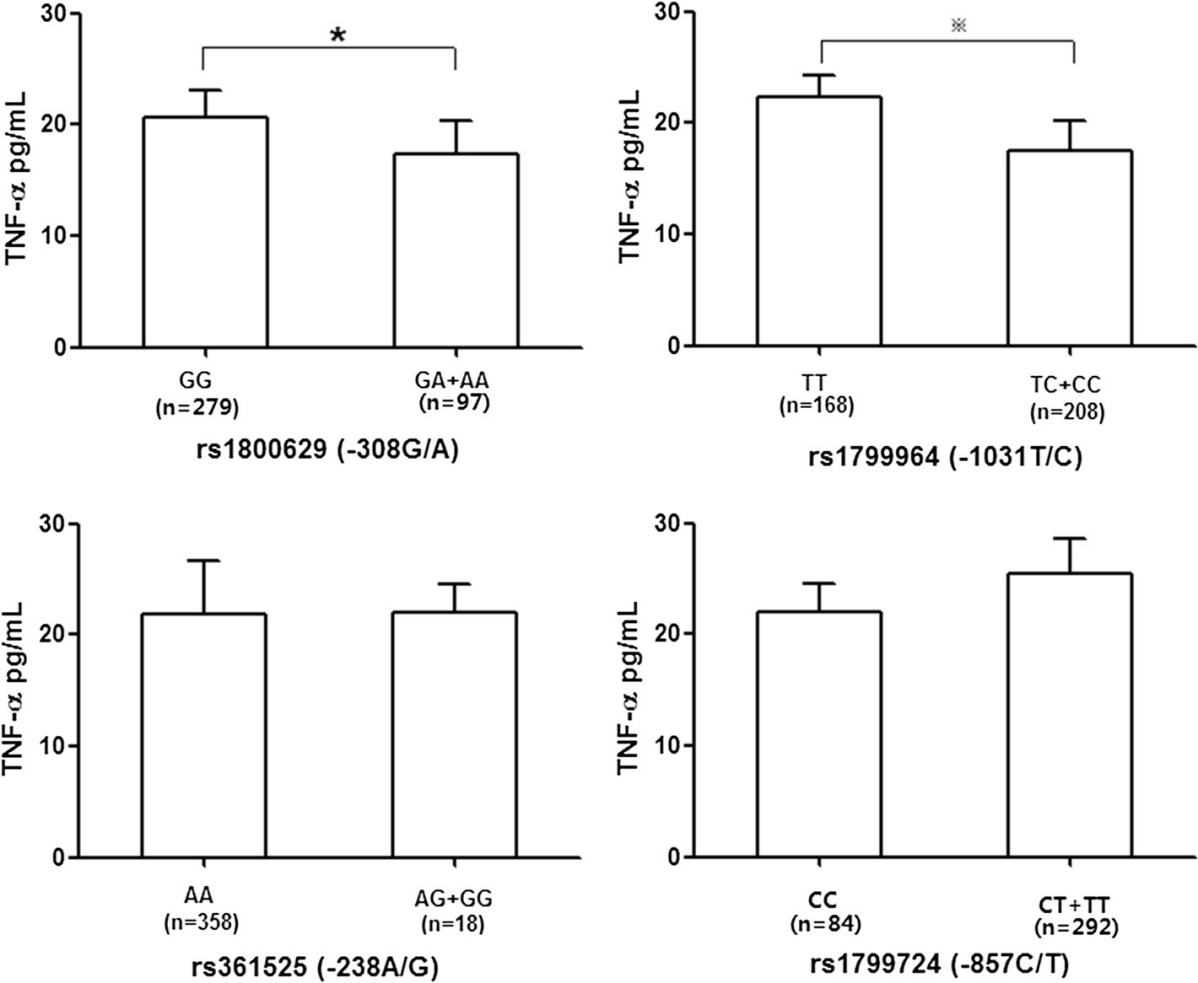
**Genetic variants of TNF-α promoter in relation to serum TNF-α level.** The data are presented as mean ± SEM. *P <0.05, ^※^*P* <0.01.

The relationship between TNF-α promoter polymorphisms and its serum concentration was analyzed subsequently in 376 unrelated individuals. As shown in Figure 3, the genotype TC and CC of rs1799964 and GA and AA of rs1800629 are associated with decreased serum TNF-α levels (*P* <0.01 and *P* <0.05, respectively). No association was observed between the other two SNPs (rs351525 and rs1799724) and serum TNF-α level in our populations. Combined analyses revealed a lower serum TNF-α level in subjects with -308G/A, -857C/T, and -1031T/C combination carriers than those with wild type (*P* <0.05) (Additional file 1: Figure S4). A similar trend was also observed in other combinations of TNF-α polymorphisms but this trend was no significant in our study.

### TNF-α level, cardiovascular risk factors, and risk of stroke

**Figure 4:**
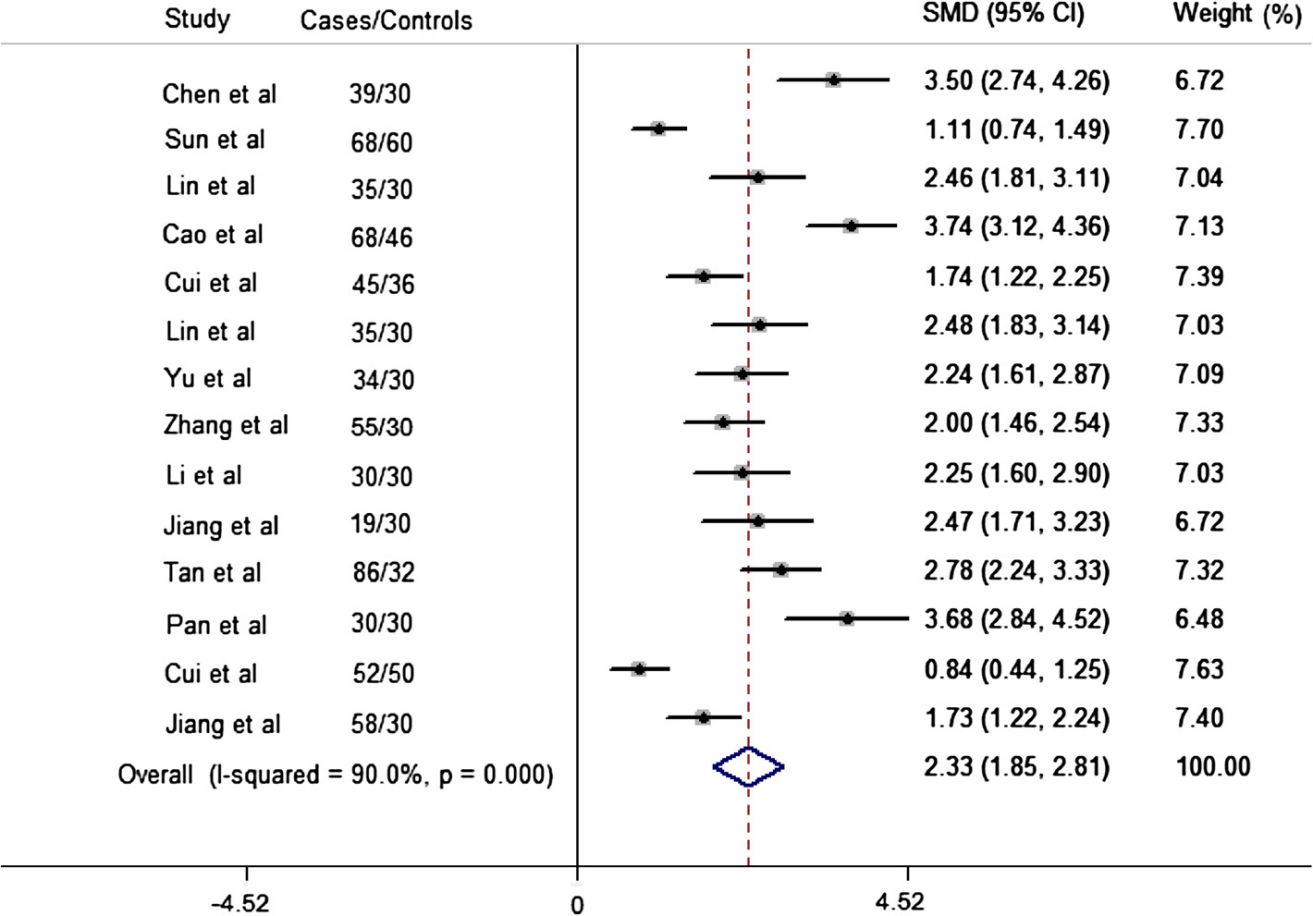
**Forest plot of the standardized mean difference (SMD) and 95% CIs of the serum TNF-α level between stroke patients and control subjects**.

We analyzed the relationship between TNF-α level in serum and patients with acute ischemic stroke in Chinese Han population. The patients consecutively admitted first-ever ischemic stroke within the first 24 h from onset were included in our meta-analysis. Details of patients’ characteristics included in our meta-analysis are covered in Additional file 1: Table S6. Meta-analysis results (Figure 4) of 14 studies (671 cases and 534 control subjects) demonstrated that ischemic stroke patients have higher serum TNF-α level than the control subjects (SMD = 2.33, 95% CI 1.85 to 2.81), although significant heterogeneity was present (I^2^ =0.9, *P*_heterogeneity_ < 0.0001). The Egger’s test and Begg’s funnel plot were applied for comparison to assess the publication bias of the literatures, and a possibility of publication bias for this test was observed (*P* = 0.001) (Additional file 1: Figure S2B). Sensitivity analyses indicated that there was also no significant trend that the overall result was influenced by any individual study (Additional file 1: Figure S3B).

**Figure 5:**
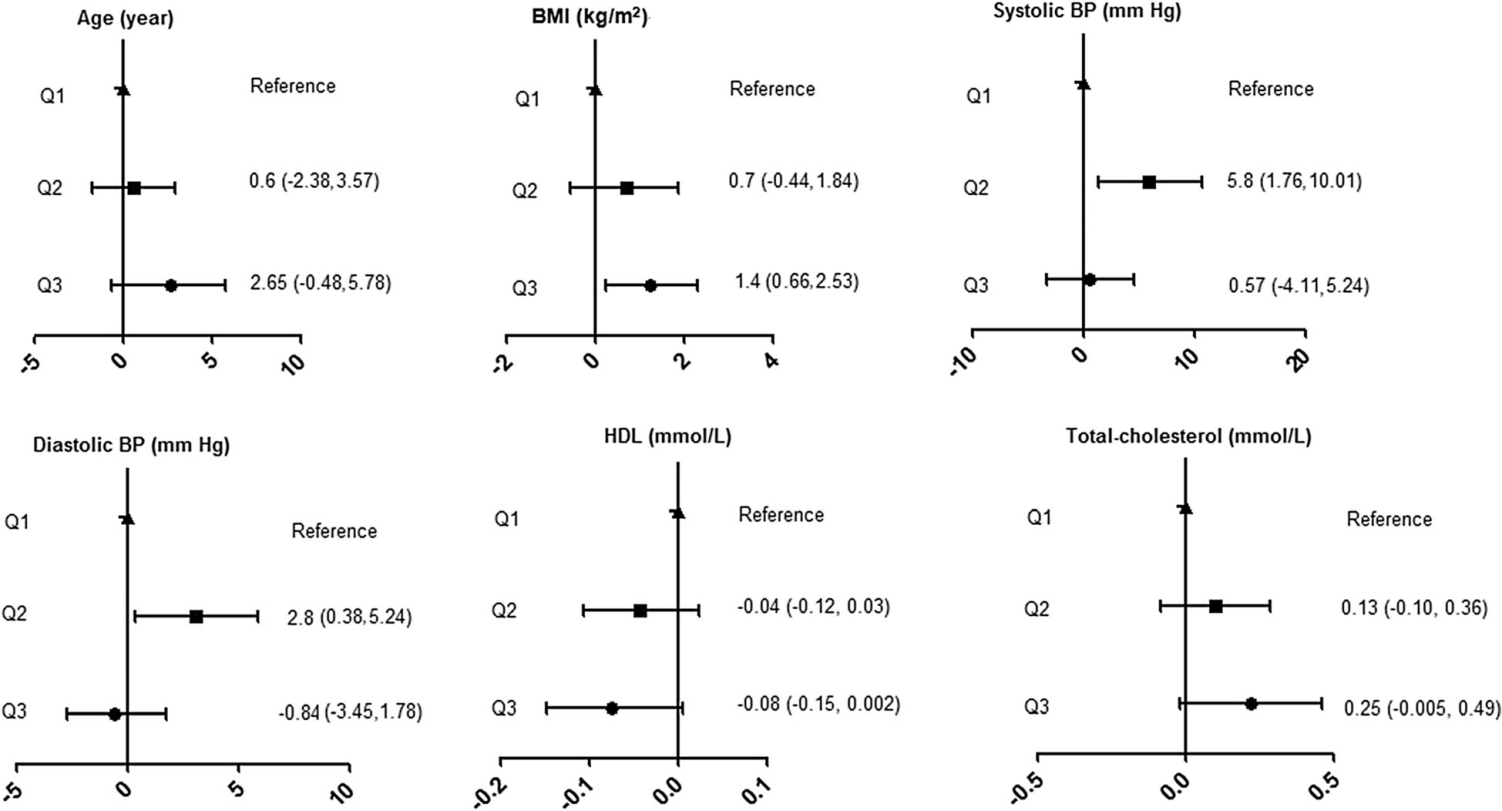
**Mean differences in cardiovascular traits by quartiles (Q) of serum TNF-α level.** Subjects from the bottom quartile (Q1) were used as the reference group. BMI, body mass index; BP, blood pressure.

Next, we assessed the association between serum TNF-α level and traditional cardiovascular risk factors. As shown in Figure 5, we can see the age, BMI, and total cholesterol were higher by quartiles of increasing TNF-α level. The similar trend for blood pressure was only found in Q1 and Q2. Conversely, there was an inverse association between quartiles of serum TNF-α level and HDL cholesterol. However, this trend was not statistically significant.

### Effects of polymorphisms on *in vitro* activity of the TNF-α promoter and interact function study

**Figure 6:**
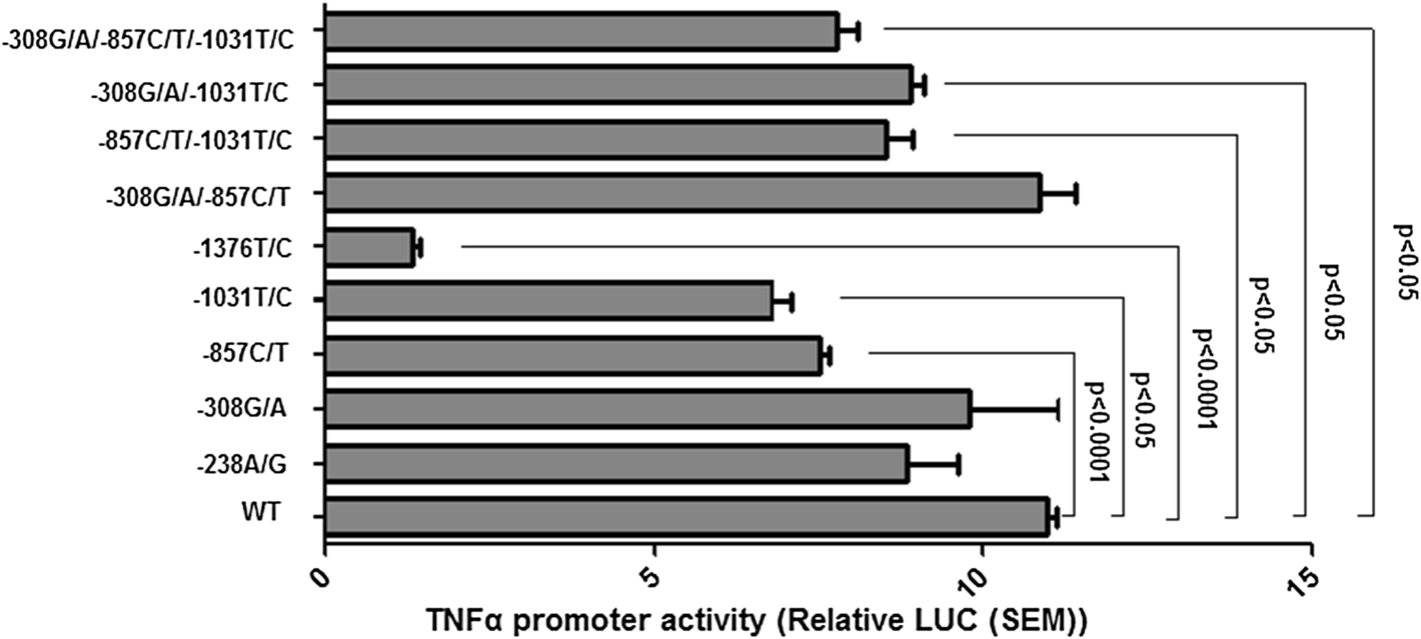
**Expression studies of TNF-α promoter constructs carrying -238A/G, -308G/A, -857C/T, -1031T/C, and -1376T/C and potential interact together between -308G/A, -857C/T, and -1031T/C polymorphisms.** TNF-α promoter activity is expressed as fold increase of RLU relative to pGL3 basic. Mutant construct compared to the wild-type construct for each comparison. Values are mean ± SE of three independent experiments each corresponding to at least five replicates.

To test whether the polymorphisms in the TNF-α promoter region were functionally important for the regulation of TNF-α transcription, we performed functional analyses comparing the activities of four SNPs (MAF > 0.02) -308G/A, -238A/G, -857C/T, -1031T/C, and a novel SNP -1376T/C alleles in HepG2 cells. The -863C/A was in strong LD with -1031T/C and was not applied for the further functional analysis. As shown in Figure 6, reporter gene expression for the -857T, -1031C, -1376C allele was significantly reduced compared to the wild type allele (*P* <0.0001, *P* <0.05, and *P* <0.0001, respectively).

Given the frequency of the polymorphism and to test whether these common SNPs (MAF >0.05) may act cooperatively, another four constructs were made for transient transfection, including -308G/A / -857C/T, -308G/A / -1031T/C, -857C/T / -1031T/C, -308G/A / -857C/T / -1031T/C. We found that the relative luciferase activity of the rare allele combination -308G/A / -1031T/C, -857C/T / -1031T/C, -308G/A / -857C/T / -1031T/C were significantly lower compared to wild-type ones (*P* <0.05, *P* <0.05, *P* <0.05, respectively) (Figure 6), which suggest that the functional polymorphisms may act in a cooperative manner of interact together to determine the overall activity of the TNF-α gene promoter.

## Discussion

The present systematic study examined the polymorphism of TNF-α gene promoter, circulating TNF-α levels, and traditional cardiovascular risk factor for ischemic stroke in the Chinese Han population. Our findings show that the polymorphisms of TNF-α promoter may function as an important risk factor for ischemic stroke and our meta-analysis data confirmed that patients with acute ischemic stroke compared to controls without stroke have significantly higher median serum levels of TNF-α. TNF-α level was also positively associated with some established traditional cardiovascular risk factors, age, BMI, total cholesterol, blood pressure, and inversely with HDL in our study. Additional functional analyses showed that variants in TNF-α gene promoter region may interact together to determine the overall activity of the TNF-α gene promoter. The flow chart illustrating our study process was shown in Additional file 1: Figure S5.

SNPs at the promoter region of the TNF-α gene have been commonly studied. Resequencing of 96 unrelated Chinese Han individuals revealed high linkage disequilibrium between the −863 C/A and the -1031T/C and no other SNPs have any significant LD with each other. But complete allelic associations were observed between the -238G/A and the -1031T/C, and between the −863 C/A and the -1031T/C in Europeans [10]. The partial LD was also observed between the TNF-α -1031T/C and TNF-α -863C/A polymorphisms (D’ =0.75) in an Indian population and the similar results were also found between -308G/A and -1031T/C and -308G/A and -863C/A in this study (D’ = 0.74 and D’ =0.73, respectively) [28]. In addition, five polymorphisms (−308G/A, -238G/A, -857C/T, -863C/A, -1031T/C) located in the human TNF-α gene promoter have been identified and the allele frequencies of these polymorphisms observed in this study in a Chinese Han population are quite distinct from those reported by Skoog *et al.* in a European population [10]. The different allelic frequency and partial LD may be due to the differences in the genetic backgrounds and these differences also indicated that these polymorphisms of TNF-α promoter may act on different function in different ethnic group.

In our next case–control study and meta-analysis, we confirmed the significant association between TNF-α promoter variant -308G/A and stroke in the Asian population. Recently, several GWASs for stroke have been reported [29-34], but most of these study populations were of European origin and they did not detect the association of rs1800629 in TNF-α with stroke. Indeed, the similar negative results were obtained from the European populations reported by Freilinger *et al*. [35]. Recently, due to the small sample size in stage 1 of GWAS screening (131 cases and 135 controls), the GWASs performed in the Japanese population [31] did not have sufficient power to observe a positive association. These data imply that the variant rs1800629 might have different effects on stroke (or other biological and pathological processes) among different populations; however, up to now, no GWASs results for stroke were reported in Chinese Han populations.

Inflammatory processes have increasingly been shown to be involved in the pathogenesis of cerebrovascular diseases, including ischemic stroke and cerebral hemorrhage. We demonstrate elevated serum TNF-α level was associated with higher risk of ischemic stroke in the Chinese Han population. By contrast, the TNF-α was not associated with risk of stroke in a British prospective study [14]. Another meta-analysis also confirmed that TNF-α was linked to a 1.6-fold increase in ischemic stroke risk in adult Asian subjects but had no effect on European ancestry [13]. Likewise, we found a weakly association between TNF-α level and different genotypes of -308G/A. Indeed, we cannot formally exclude the possibility that other functional SNPs, linked to the -308G/A, may influence the expression of TNF-α or other functional SNPs may interact together with -308G/A polymorphism to play a different role in different pathological processes. On the other hand, there was no clear signal for an effect of TNF-α gene variant on cardiovascular risk factors but TNF-α level itself was correlated with a range of baseline characters in our study subjects, and the findings are consistent with those of the previous reports in other ethnic groups [14,21,36]. However, we can see an inverse correlation between Q3 of TNF-α level and blood pressure, and this may be due to the smaller cohort size in our study.

To date, there is no consensus regarding the functional significance of TNF gene polymorphisms. A number of explanations for this discrepancy have been offered including cell types, stimulants, and reporter gene constructs. Therefore, these polymorphisms may serve as markers for additional polymorphisms in the TNF-α locus or neighboring genes that may influence disease severity or functional activity. In this study, we provided additional evidence that limited data reported so far do not provide strong evidence in favor of a physiological role of -308G/A and -238G/A in the regulation of TNF-α promoter activity. We also detected a -1031C allele had a significantly reduced luciferase activity compared to -1031 T allele which had a strong partial LD with -863C/A polymorphism and the similar results were reported by Skoog *et al*. [10]. Specifically, functional study indicated a novel mutation -1376T/C identified in our study may have a large effect on the TNF-α promoter activity. However, this rare mutation need to be verified in a larger population and assayed in other ethic subjects. In particular, our results show, for the first time, the polymorphisms of TNF-α promoter may act in a cooperative manner of interact together to determine the overall activity of the TNF-α gene promoter. Therefore, the new potential transcription factor and regulation mechanism needed to be elucidated in the future.

Our conclusions must be interpreted in the context of important potential limitations of our study. Not all variants at the TNF-α gene were assessed in this study. Complete sequencing will be necessary for systematic identification of potentially causative mutations in TNF-α whole gene function region. Another limitation of the present study is the relatively small sample size used for TNF-α level analysis, which may give rise to false associations by chance (type one error), or may fail to detect true differences. Serum TNF-α levels are affected by multiple environmental, genetic factors and their interactions. Although we have observed the effects of several metabolism characters and SNP in TNF-α on serum levels in this study, there are still many other correlative environmental and genetic factors need to be determined. Our meta-analysis results must be viewed with careful because of potential biases. Perhaps because positive results have a better chance of being accepted for publication than small studies with non-significant or negative findings. Therefore, conclusions based on these published work might be misleading [37]. Finally, it is important to confirm these findings in prospective cohort studies both in Chinese Han populations and other ethnic groups.

To conclude, this may be the first systematic and comprehensive analysis of TNF-α and stroke especially in an Asian population. Our results strongly support the involvement of TNF-α in the pathogenesis of stroke, and may have potentially important scientific, clinical, and public health implications.

## Competing interests

The authors declare that they have no competing interests.

## Authors’ contributions

GC carried out the molecular genetic studies, participated in the sequence alignment and drafted the manuscript. HW and RL participated in the sequence alignment and was involved in critically revising the manuscript for importand intellectual content. ZL, YW and RH participated in the design of the study and performed the statistical analysis. HD and DW conceived of the study, and participated in its design and coordination. All authors read and approved the final manuscript.

## Funding

This work was supported by ‘973’ projects (No. 2012CB518004, 2012CB517801) and ‘863’ project (No. 2012AA02A510).

## Supplementary Material

Additional file 1 **Table S1.** Sequences of PCR primers used for amplification and sequencing of TNF-α promoter. **Table S2.** TaqMan Primer and Probe Sequences. **Table S3.** Oligonucleotides used to create the site mutation. **Table S4.** Frequency of TNF-α promoter polymorphisms. **Table S5.** Characteristics of eligible studies included in the meta-analysis. **Table S6.** TNF-α levels of individual studies included in the meta-analysis. **Figure S1.** LD Structure and Haplotype Blocks of the TNF-α promoter. **Figure S2.** Funnel plot of selected studies. **Figure S3.** Sensitivity analyses of selected studies. **Figure S4.** Serum TNF-α concentration in corresponding with different combinations of TNF-α promoter polymorphisms. **Figure S5.** Flow chart illustrating our study process. Click here for file
