# Supplementary material for: Polymorphism of tumor necrosis factor alpha (TNF-alpha) gene promoter, circulating TNF-alpha level, and cardiovascular risk factor for ischemic stroke
Source: J Neuroinflammation. 2012 Oct 10;9:235. doi: 10.1186/1742-2094-9-235 (PMC3521196; doi:10.1186/1742-2094-9-235)
Supplement: Additional file 1 — Table S1. Sequences of PCR primers used for amplification and sequencing of TNF-α promoter. Table S2. TaqMan Primer and Probe Sequences. Table S3. Oligonucleotides used to create the site mutation. Table S4. Frequency of TNF-α promoter polymorphisms. Table S5. Characteristics of eligible studies included in the meta-analysis. Table S6. TNF-α levels of individual studies included in the meta-analysis. Figure S1. LD Structure and Haplotype Blocks of the TNF-α promoter. Figure S2. Funnel plot of selected studies. Figure S3. Sensitivity analyses of selected studies. Figure S4. Serum TNF-α concentration in corresponding with different combinations of TNF-α promoter polymorphisms. Figure S5. Flow chart illustrating our study process. [file 1742-2094-9-235-S1.doc]

**Supplementary Materials**

| **Supplementary Table1. Sequences of PCR primers used for amplification and sequencing of TNF-α promoter** | | | | |
| --- | --- | --- | --- | --- |
| **Region (bp)**^a^ | **Forward primers(5’ →3’)** | **Reverse primers(5’ →3’)** | **Size (bp)** |  |
| –1420 ～ –2000 | TTCAAGACCTTCTCCCCATTC | GAGGTTTATTGGGCTTCATCG | 580 |  |
| –1520 ～ –960 | CCAGAAGGCAGGGAAAGG | TTAAACGTCCCCTGTATTCCAT | 560 |  |
| –1080 ～ –469 | TGACCACAGCAATGGGTAGGA | CCCCTCAAAACCTATTGCCTC | 611 |  |
| +30 ～ –560 | GAAGTTAGAAGGAAACAGACCACAG | CACGTCCCGGATCATGCT | 590 |  |

^a^The amplification Fragments of the TNF-α promoter are numbered relative to transcription start site.

| **Supplementary Table 2. TaqMan Primer and Probe Sequences** | | | |
| --- | --- | --- | --- |
|  | **Primer (5’ →3’)** | **Allele** | **Allelic Probe** |
| rs1800629 | Forward GCCACTGACTGATTTGTGTGTAGG | C | FAM-CCGTCCCCATGCC-MGB |
|  | Reverse GGAAGTTAGAAGGAAACAGACCACAG | T | HEX-CCGTCCTCATGCC-MGB |
| rs1799964 | Forward TCTCCCCAGAGGTCTCCTGTAA | A | FAM-TTTCCTTCATCTTCTCA-MGB |
|  | Reverse GCTTCAGGGATATGTGATGGACT | G | HEX-TTTCCTTCGTCTTCTC-MGB |
| rs361525 | Forward ACCCCTCACACTCCCCATC | C | FAM-CCTGCTCCGATTC-MGB |
|  | Reverse ACGGGGTTCAGCCTCCA | T | HEX-CCCTGCTCTGATTC-MGB |
| rs1799724 | Forward CAGGGCTATGGAAGTCGAGTATG | C | FAM-CCCTTAACGAAGACAG-MGB |
|  | Reverse CTCAGGGCCCCAGTGTGT | T | HEX-CCCTTAATGAAGACAGG-MGB |

FAM indicates FAM fluorescence reporter, HEX indicates HEX fluorescence reporter.

| **Supplementary Table 3. Oligonucleotides used to create the site mutation** | |
| --- | --- |
| Oligonucleotide name | Oligonucleotide sequence |
| Mutation-rs361525-forward | 5' ACCCCCCTCGGAATCAGAGCAGGGAGGATG 3' |
| Mutation-rs361525-reverse | 5' CATCCTCCCTGCTCTGATTCCGAGGGGGGT 3' |
| Mutation-rs1799724-forward | 5' GGACCCCCCCTTAATGAAGACAGGGCCATG 3' |
| Mutation-rs1799724-reverse | 5' CATGGCCCTGTCTTCATTAAGGGGGGGTCC 3' |
| Mutation-rs1799964-forward | 5' AGGAGAAGCTGAGAAGACGAAGGAAAAGTCAGGGTC 3' |
| Mutation-rs1799964-reverse | 5' GACCCTGACTTTTCCTTCGTCTTCTCAGCTTCTCCT 3' |
| Mutation-rs1800629-forward | 5' GTTTTGAGGGGCATGAGGACGGGGTTCAGC 3' |
| Mutation-rs1800629-reverse | 5' GCTGAACCCCGTCCTCATGCCCCTCAAAAC 3' |
| Mutation-(-1376)-forward | 5' GGGAGTGAGAACTTCTCAGTCTATCTAAGGAAT 3' |
| Mutation-(-1376)-reverse | 5' ATTCCTTAGATAGACTGAGAAGTTCTCACTCCC 3' |

| **Supplementary Table 4. Frequency of TNF-α promoter polymorphisms** | | | | |
| --- | --- | --- | --- | --- |
| **dbSNP name^a^** | **Function** | **Position^e^** | **Alleles^f^** | **MAF^g^** |
| rs1799964 | promoter | -1031 | T/C | 0.068 |
| rs1799724 | promoter | -857 | C/T | 0.109 |
| rs1800630 | promoter | -863 | C/A | 0.068 |
| rs1800629 | promoter | -308 | G/A | 0.021 |
| rs4248158 | promoter | -986 | C/T | 0.016 |
| rs361525 | promoter | -238 | A/G | 0.021 |
| rs3093547 | promoter | -1671 | T/A | 0.016 |
| –1376T/C^b^ | promoter | -1376 | T/C | 0.005 |
| ^a^ If present in NCBI database; | | | | |
| ^b^SNPs are numbered relative to transcription start site; | | | | |
| ^e^ Position is base-pair location in NCBI build 36.1; | | | | |
| ^f^ With major allele given first and minor allele given second; | | | | |
| ^g^MAF: minor allele frequency. | | | | |

| **Supplementary Table 5. Characteristics of eligible studies included in the meta-analysis** | | | | | | | |  |  |
| --- | --- | --- | --- | --- | --- | --- | --- | --- | --- |
| Study | Year |  | Study | Cases | | Controls | | Ethnicity | Phenotypes |
|  |  | No.of Cases/Controls | Design | Mean Age | Male% | Mean Age | Male% |  |  |
|  |  |  |  | (year) |  | (year) |  |  |  |
| Lee et al | 2004 | 152/165 | C C | 60.9 | 55.9 | 59.1 | 47.3 | South Korea | IS |
| Um and Kim et al | 2004 | 366/610 | C C | 61 | 49.2 | 62.2 | 48.2 | South Korea | CI |
| Bo et al | 2004 | 42/31 | C C | 56 | 59.5 | 55 | 58 | Han | CI |
| Ok Joon Kim et al | 2010 | 237/216 | C C | 61.68 | 58.2 | 60.32 | 52.3 | South Korea | IS |
| Ma et al | 2003 | 1122/1123 | C C | 62.8 | 61.4 | 62.6 | 61.6 | Han | IS |
| Tong et al | 2010 | 648/648 | C C | 60.21 | 58.49 | 60.21 | 58.49 | Han | IS |
| Tong et al | 2010 | 100/100 | C C | 64.12 | 58 | 63.18 | 58 | Uyghur | IS |
| Li et al | 2009 | 97/141 | C C | 64.8 | 53.6 | 62.71 | 49.6 | Han | CI |
| Jae-Young Um et al | 2003 | 294/581 | C C | 61 | 49.3 | 62.2 | 48.2 | South Korea | CI |
| Jae-Young Um et al | 2005 | 212/610 | C C | 46.9 | 52.4 | 62.2 | 48.2 | South Korea | CI |
| Karahan et al | 2005 | 86/83 | C C | 5.5 | 53.5 | NA | 43.4 | Turkish | IS |
| Sultana S et al | 2011 | 238/226 | C C | 53.72 | 68.9 | 54.06 | 53.5 | india | IS |
| Wang et al | 2009 | 1163/1471 | C C | 59.3 | 60.3 | 61.1 | 60.1 | Han | IS |
| IS, ischemic stroke; CI, cerebral infarction. | | | | | | | | | |

| **Supplementary Table 6. TNF-α levels of individual studies included in the meta-analysis** | | | | | | | | | | | |  | |  |
| --- | --- | --- | --- | --- | --- | --- | --- | --- | --- | --- | --- | --- | --- | --- |
| Study | Year |  | Case | |  | |  | | Control | | |  | |  |
|  |  | NO. | Mean Age | Male% | TNF-α | NO. | | Mean Age | | Male% | TNF-α | | Ethnicity | |
|  |  |  | (year) |  | level |  | | (year) | |  | level | |  | |
| Chen et al | 2005 | 39 | 46-70^a^ | 26/39 | 59.38±14.37 | 30 | | NA | | 18/30 | 18.71±6.38 | | Han | |
| Sun et al | 2006 | 68 | 69 | no | 0.99±0.15 | 60 | | 65 | | NA | 0.77±0.24 | | Han | |
| Lin et al | 2009 | 35 | 32-78^a^ | 24/35 | 33.68±15.24 | 30 | | NA | | NA | 5.8±2.73 | | Han | |
| Cao et al | 2002 | 68 | 74.32 | 40/68 | 1.58±0.39 | 46 | | 63.41 | | 24/46 | 0.39±0.16 | | Han | |
| Cui et al | 2003 | 45 | 61.2 | 26 | 125.8±40.2 | 36 | | 62.4 | | 21 | 70.3±16.6 | | Han | |
| Lin et al | 2009 | 35 | 56.9 | 24 | 33.68±15.24 | 30 | | NA | | NA | 5.80±1.23 | | Han | |
| Yu et al | 2004 | 34 | 68 | 17 | 2.4±0.7 | 30 | | 55 | | 18 | 1.1±0.4 | | Han | |
| Li et al | 2003 | 30 | 66.23 | 20 | 2915±656.42 | 30 | | 63.07 | | 17 | 1411.67±678.95 | | Han | |
| Zhang et al | 2007 | 55 | 73.49 | 62 | 0.12±0.01 | 30 | | 70 | | 70 | 0.07±0.04 | | Han | |
| Jiang et al | 2007 | 19 | 58 | 11 | 1.98±0.68 | 30 | | 56 | | 19 | 0.85±0.23 | | Han | |
| Tan et al | 2010 | 86 | 63.4 | 52 | 21.34±5.76 | 32 | | 62.1 | | 18 | 7.26±2.18 | | Han | |
| Pan et al | 2007 | 30 | 66.8 | 18 | 57.78±14.79 | 30 | | 64.2 | | 20 | 16.38±5.83 | | Han | |
| Cui et al | 2008 | 52 | 60 | 30 | 21.11±6.12 | 50 | | 59 | | 30 | 16.08±5.82 | | Han | |
| Jiang et al | 2008 | 58 | 61 | 32 | 1.78±0.64 | 30 | | 58 | | 18 | 0.85±0.23 | | Han | |
| Data were expressed as mean±SD | | | | | | | | | | | | | | |
| ^a^Only age range were available | | | | | | | | | | | | | | |
| NA: Date was not available | | | | | | | | | | | | | | |

**Figure Legends**

**Supplementary Figure 1. LD Structure and Haplotype Blocks of the TNF-α promoter.** Linkage disequilibrium (D′) for identified polymorphisms in TNF-α, as generated by Haploview 4.0 from the genotype data on 96 random control subjects. Haplotype blocks derived from these genotypes using the solid spine linkage disequilibrium setting are outlined in black.

**Supplementary Figure 2. Funnel plot of selected studies.** Funnel plot describing the relationship between effect size and standard error (SE) of effect with possible missing studies imputed. Vertical line, mean effect size; dashed line, pseudo 95% confidence limit.

**Supplementary Figure 3.** **Sensitivity analyses of selected studies.** Each line showed the recalculated pooled relative risk of remaining studies by omitting one study listed in the left volume a time.

**Supplementary Figure 4. Serum TNF-α concentration in corresponding with different combinations of TNF-α promoter polymorphisms.** 0, wild type; 1, -308G/A and -857C/T; 2, -308G/A and -1031T/C; 3, -857C/T and -1031T/C; 4, -308G/A, -857C/T and -1031T/C. The data are presented as mean±SEM. *P<0.05

**Supplementary Figure 5. Flow chart illustrating our study process.**

**Supplementary Figure 1.**
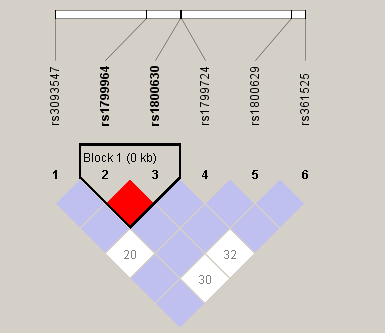


**Supplementary Figure 2.**
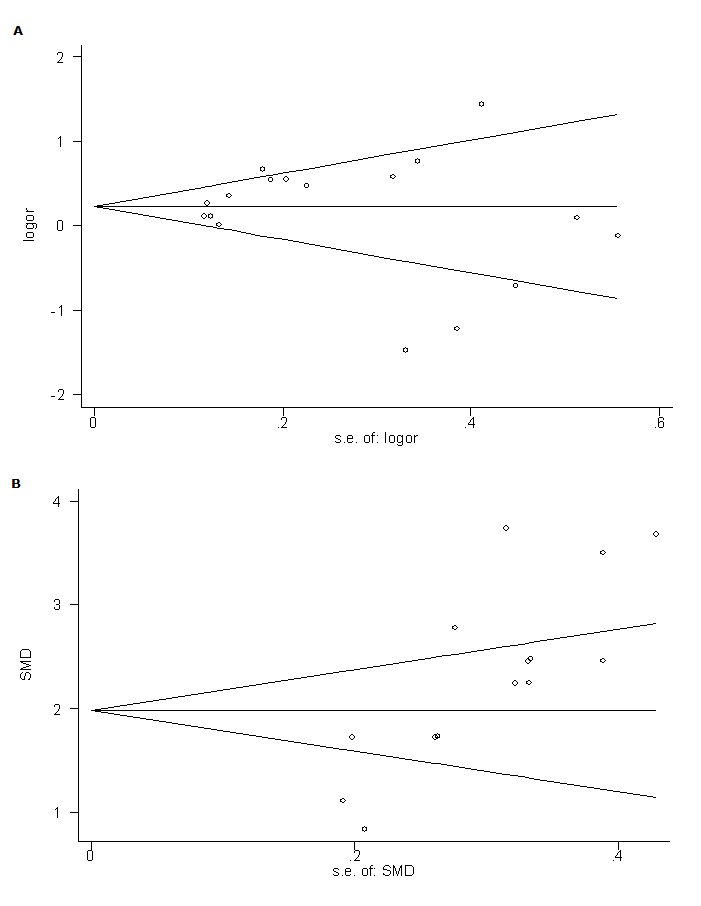


**Supplementary Figure 3.**
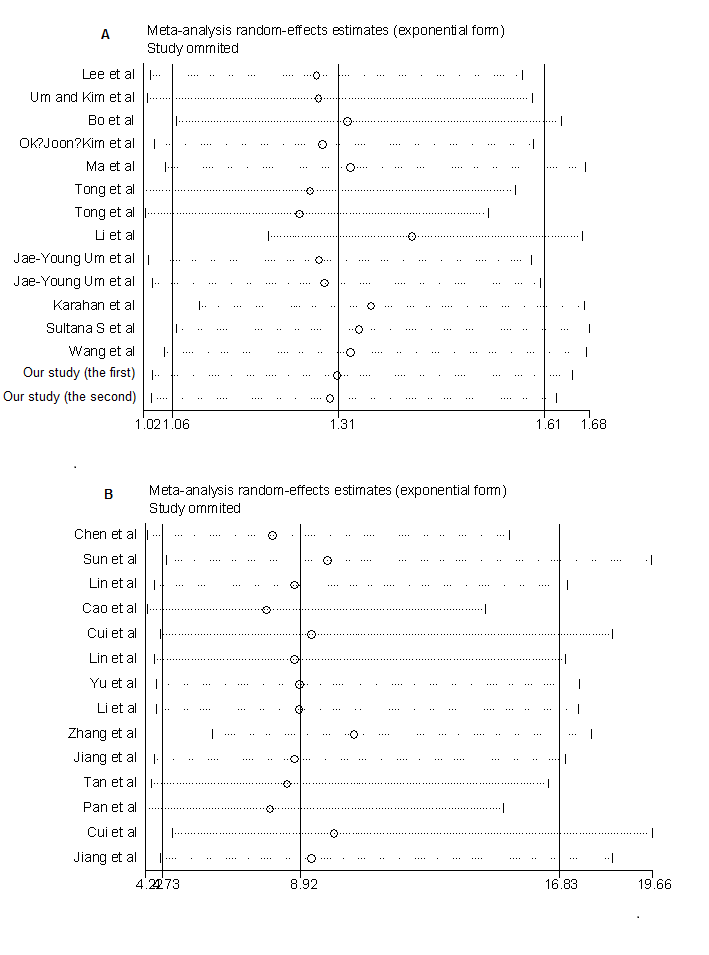


**Supplementary Figure 4.**


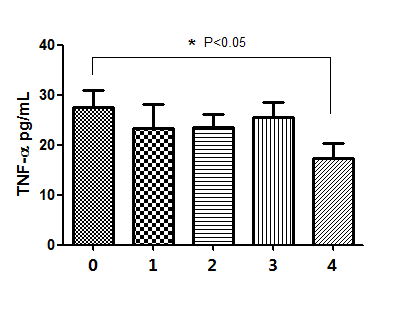


**Supplementary Figure 5.**

**
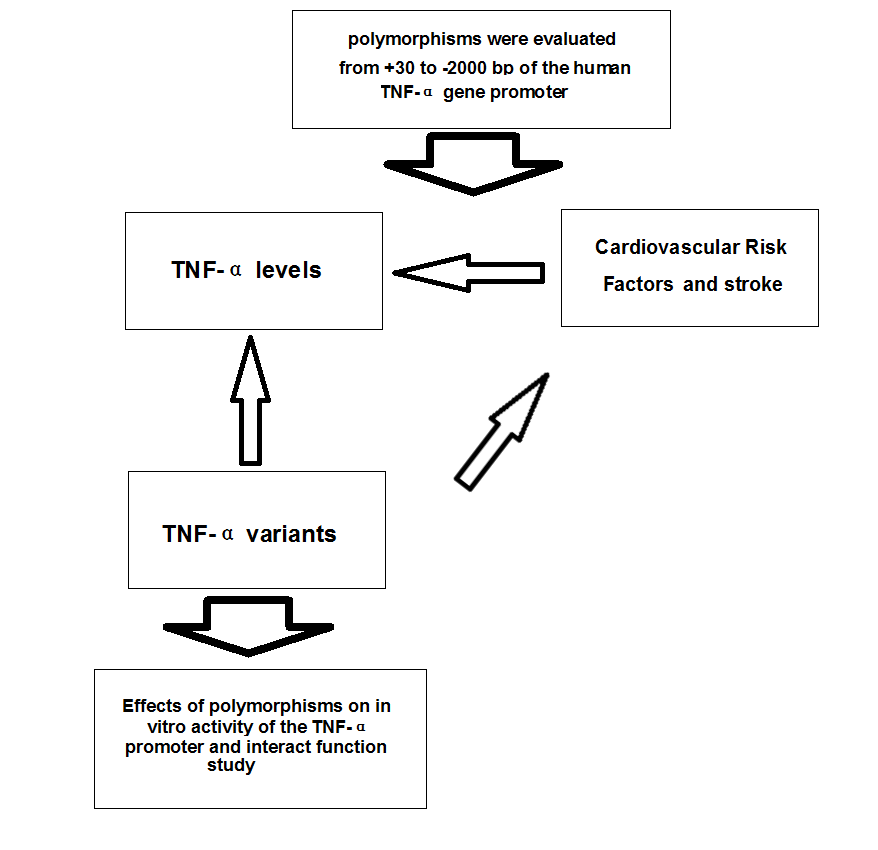
**

**References**

1. Lee BC, Ahn SY, Doo HK, Yim SV, Lee HJ, Jin SY, Hong SJ, Lee SH, Kim SD, Seo JC, et al: **Susceptibility for ischemic stroke in Korean population is associated with polymorphisms of the interleukin-1 receptor antagonist and tumor necrosis factor-alpha genes, but not the interleukin-1beta gene.** *Neurosci Lett* 2004, **357:**33-36.

2. Um JY, Kim HM: **Tumor necrosis factor alpha gene polymorphism is associated with cerebral infarction.** *Brain Res Mol Brain Res* 2004, **122:**99-102.

3. Xuegong Bo ZC: **Correlation between tumor necrosis factor alpha gene polymorphism and cerebral infarction.** *J New Med* 2004, **14:**111-113.

4. Kim OJ, Lee JH, Choi JK, Oh SH, Hong SH, Oh D, Kim NK: **Association between tumor necrosis factor-alpha (-308G-->A and -238G-->A) polymorphisms and homocysteine levels in patients with ischemic strokes and silent brain infarctions.** *Cerebrovasc Dis* 2010, **30:**483-490.

5. Liyuan Ma WW, Lisheng Liu: **The polymorphisms of ischemic stroke related genes in Chinese population.** *Chinese Journal of Hypertension* 2003, **11:**116-120.

6. Tong Y, Geng Y, Xu J, Wang Z, Zhang Y, Lin L, Zhang R, Deng P, Li Y, Hou W, et al: **The role of functional polymorphisms of the TNF-alpha gene promoter in the risk of ischemic stroke in Chinese Han and Uyghur populations: Two case-control studies.** *Clin Chim Acta* 2010, **411:**1291-1295.

7. Mingshao Li YC, Shengqiang Chen: **Relationship of TNF-α -238 and TNF-α -308 polymorphisms with atherosclerosis cerebral infarction.** *Chin J Biomed Eng* 2009, **15:**98-102TNF-α.

8. Um JY, An NH, Kim HM: **TNF-alpha and TNF-beta gene polymorphisms in cerebral infarction.** *J Mol Neurosci* 2003, **21:**167-171.

9. Um JY, Lee JH, Joo JC, Kim KY, Lee EH, Shin T, Hong SH, Kim HM: **Association between tumor necrosis factor-alpha gene polymorphism and Sasang constitution in cerebral infarction.** *Am J Chin Med* 2005, **33:**547-557.

10. Karahan ZC, Deda G, Sipahi T, Elhan AH, Akar N: **TNF-alpha -308G/A and IL-6 -174 G/C polymorphisms in the Turkish pediatric stroke patients.** *Thromb Res* 2005, **115:**393-398.

11. Sultana S, Kolla VK, Jeedigunta Y, Penagaluru PK, Joshi S, Rani PU, Reddy PP: **Tumour necrosis factor alpha and interleukin 10 gene polymorphisms and the risk of ischemic stroke in south Indian population.** *J Genet* 2011, **90:**361-364.

12. Wang X, Cheng S, Brophy VH, Erlich HA, Mannhalter C, Berger K, Lalouschek W, Browner WS, Shi Y, Ringelstein EB, et al: **A meta-analysis of candidate gene polymorphisms and ischemic stroke in 6 study populations: association of lymphotoxin-alpha in nonhypertensive patients.** *Stroke* 2009, **40:**683-695.

13. Fangmei Chen QY: **Changes in serum IL-6, TNF-α and adhesion molecules in patients with acute cerebral infarction.** *Sect Clin Biochem & Lab Med Foreign Med Sci* 2005, **26:**689-693.

14. Jianfang Sun JS: **Changes in serum CRP, TNF-α and IL-6 in elderly patients with acute stroke.** *Chin J geriatr* 2006, **25:**195-196.

15. Jiuzuo Lin KM, Haixia Zhang, Qingzuan Kong, Riming Yuan, Zhenwei Wang, Shunxiang Liu: **The predictive value of early detection of serum tumor necrosis factor alpha and interleukin-6 for progressive cerebral infarction.** *Chin J Postgrad Med* 2009, **32:**7-9.

16. Yin Cao LQ, Xiumei Wu: **The change of plasma tuma necrosis factor-α concentration in patients with acute cerebral infarction.** *Med J CASC* 2002, **4:**17-18.

17. Jingbin Cui JH, Lin Guo, Junping Wang: **Clinical signifieance of serum interleukin-6 and tumor necrosis factor-α in patients with acute cerebral infarction.** *Hennan Medical Research* 2003, **12:**41-42.

18. Jiuzuo Lin KM, Haixia Zhang, Qingzuan Kong, Riming Yuan, Zhenwei Wang, Shunxiang Liu: **Change and its significance of serum TNF-α and IL-6 in acute cerebral infarction.** *JOURNAL OF ZHE JIANG UNIVERSITY (MEDICAL SCIENCES)* 2010, **39:**415-418.

19. Peng Yu YS: **Study on the change of TNF-α in serum of the critical stroke patients.** *Chn Jrnl of Crbrvsclr Dieases* 2004, **1:**125-126.

20. Mingshan Li BL: **Serum level of soluble selectin-E, tumor necrosis factor levels of determination and significance of cerebral infarction.** *Clinical Focus* 2003, **18:**871-872.

21. Mingyi Zhang J, Yun Zhang, Zhihua Dai: **The effect of serum tumor necrosis factor-alpha on the prognosis of patients with acute ischemic cerebral stroke.** *Chin J Contemp Neurosurg* 2007, **7:**268-271.

22. Xiulong Jiang XZ, Huixing Lei, Qiong Cheng: **Changes and clinical significance of serum IL-6, TNF-α, S-100b protein in patiedts with progressive ischemic stroke.** *Chin J Diffie and Comp Cas* 2007, **6:**658-661.

23. Dailin Tan AC: **Acute ischemic stroke patients with serum high sensitivity C-reactive protein and levels of inflammatory cytokines and clinical significance.** *Chinese Journal of microcirculation* 2010, **20:**55-56.

24. Aiwu Pan JS, Dacheng Xu: **Changes of plasma CRP, TNF-α and IL-6 levels in patients with acute cerebral infarction.** *Clinical Education of General Practice* 2007, **5:**193-195.

25. Cui F: **Changes in serum levels of TNF-α in in patients with acute cerebral infarction.** *Journal of Hebei North University (Medical Edition)* 2008, **25:**47-48.

26. Xiulong Jiang XJ, Xu Zhang: **Changes and clinical significance of serum IL-6, TNF-α and S-100b in patients with acute cerebral infarction.** *J Neurol Neurorehabil* 2008, **5:**197-201.
